# Over-Expression of Monoacylglycerol Lipase (MGL) in Small Intestine Alters Endocannabinoid Levels and Whole Body Energy Balance, Resulting in Obesity

**DOI:** 10.1371/journal.pone.0043962

**Published:** 2012-08-28

**Authors:** Su-Hyoun Chon, John D. Douglass, Yin Xiu Zhou, Nashmia Malik, Joseph L. Dixon, Anita Brinker, Loredana Quadro, Judith Storch

**Affiliations:** 1 Department of Nutritional Sciences, Rutgers University, New Brunswick, New Jersey, United States of America; 2 Department of Food Science Rutgers, Rutgers University, New Brunswick, New Jersey, United States of America; 3 Rutgers Center for Lipid Research, Rutgers University, New Brunswick, New Jersey, United States of America; University of Cordoba, Spain

## Abstract

The function of small intestinal monoacylglycerol lipase (MGL) is unknown. Its expression in this tissue is surprising because one of the primary functions of the small intestine is to convert diet-derived MGs to triacylglycerol (TG), and not to degrade them. To elucidate the function of intestinal MGL, we generated transgenic mice that over-express MGL specifically in small intestine (iMGL mice). After only 3 weeks of high fat feeding, iMGL mice showed an obese phenotype; body weight gain and body fat mass were markedly higher in iMGL mice, along with increased hepatic and plasma TG levels compared to wild type littermates. The iMGL mice were hyperphagic and displayed reduced energy expenditure despite unchanged lean body mass, suggesting that the increased adiposity was due to both increased caloric intake and systemic effects resulting in a hypometabolic rate. The presence of the transgene resulted in lower levels of most MG species in intestinal mucosa, including the endocannabinoid 2-arachidonoyl glycerol (2-AG). The results therefore suggest a role for intestinal MGL, and intestinal 2-AG and perhaps other MG species, in whole body energy balance via regulation of food intake as well as metabolic rate.

## Introduction

Fatty acids and *sn*-2-monoacylglycerol (MG) are the major hydrolysis products of dietary TG in the intestinal lumen [1]. The MG acylation pathway plays a key role in small intestinal TG re-synthesis during fat absorption, as more than 80% of postprandial enterocyte TG formation is catalyzed by this pathway [2], [3]. Sequential acylation toward TG is initiated by monoacylglycerol acyltransferase (MGAT; EC 2.3.1.22), and completed by diacylglycerol acyltransferase (DGAT; EC 2.3.1.20). Genes encoding for both these enzymes have been identified [4]–[9] and each enzyme has several isoforms (MGAT 1, 2, and 3 and DGAT1and 2). With the exception of MGAT1, all show robust expression in small intestine [4]-[9]. The presence of redundant acylating enzymes, as well as the likelihood of serial acylation by some of the enzymes [10], [11], reflects the physiological importance of TG reesterification and chylomicron biogenesis in the small intestine.

In addition to the well-characterized esterification pathway, we recently demonstrated for the first time the presence of MG lipase (MGL; EC 3.1.1.23) gene expression in intestinal mucosa, supporting a catabolic pathway for MG in small intestinal epithelium [12], [13]. Duncan *et. al.* showed that MGL expression and activity were present in both mucosal and muscle layers of the small intestine [14]. In brain, MGL works as a primary regulator of *sn*-2-arachidonoyl glycerol (2-AG) signaling action via the endocannabinoid (EC) system [15], [16]. We showed that intestinal MGL expression and activity are significantly induced by a high fat diet but not by fasting, suggesting a potential role for MGL in dietary lipid assimilation [13]. At present, however, the functional implications of intestinal MG hydrolysis have been little explored.

In an effort to understand the function of intestinal MGL, we generated transgenic mice (iMGL mice) that overexpress MGL specifically in small intestine using the intestinal fatty acid binding protein (IFABP) promoter, and investigated the effects on intestinal lipid metabolism as well as whole body energy homeostasis. Analysis of mucosal lipid composition showed significant reduction in levels of MG but no changes in the composition of other lipid species, nor in the acute metabolism of radiolabeled oleate or *sn*-2-monoolein. Strikingly, after 3 weeks of a high fat (40% kcal) diet, iMGL mice showed a significant increase in body weight gain and body fat accumulation accompanied by hyperphagia, as well as reduced energy expenditure. These results suggest that alteration of intestinal MG metabolism results in important systemic effects on total body energy balance.

## Results

### Generation of Transgenic Mice Overexpressing Murine MGL Specifically in Small Intestine

**Figure 1:**
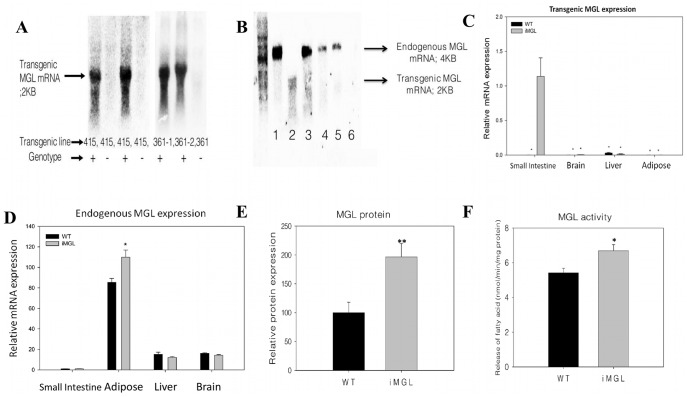
Tissue specific overexpression of transgenic MGL mRNA. (A) Overexpression of transgenic MGL transcript (2 kb) in mouse small intestine. 40 µg of total RNA was loaded onto each well, and probed with [^32^P] labeled full length coding region of mMGL cDNA. (B) Intestinal specific expression of transgenic MGL transcript by Northern analysis. 1 µg of poly A^+^ RNA was loaded onto each well, and probed with mMGL cDNA. Lane 1, Brain; lane 2, Small intestine; lane 3, Adipose tissue; lane 4, Liver; lane 5, Stomach; lane 6, Cecum. Upper bands found in lanes 1, 3, 4, and 5 corresponded to the 4 kb endogenous MGL transcript, and lower band shown in lane 2 was the 2 kb transgenic MGL transcript. (C) Intestinal specific expression of transgenic MGL transcript by qPCR analysis. A unique junction region present only in transgenic animals (MGL coding region + SV40 poly A sequence) was the target for the amplification. Data represent mean ± S.E. n = 4 per group, expression relative to iMGL small intestine. (D) Endogenous MGL mRNA expression in iMGL mice. Tissues were harvested after 3 weeks of high fat feeding. Primers were designed to amplify the 3′untranslated region of endogenous MGL mRNA sequence, which is absent in the transgenic MGL transcript.(n = 7–8 per group) (E) Relative MGL protein expression in iMGL versus wild type small intestine after 3 weeks of a high fat diet. Values are presented relative to the expression of the wild type littermates set to 100%. Data represent mean ± S.E. n = 5–8 per group. (F) MGL activity in iMGL versus wild type small intestine after 3 weeks high fat diet. n = 5–8 per group. *p<0.05, **p<0.01 versus wild type littermates.

**Figure 2:**
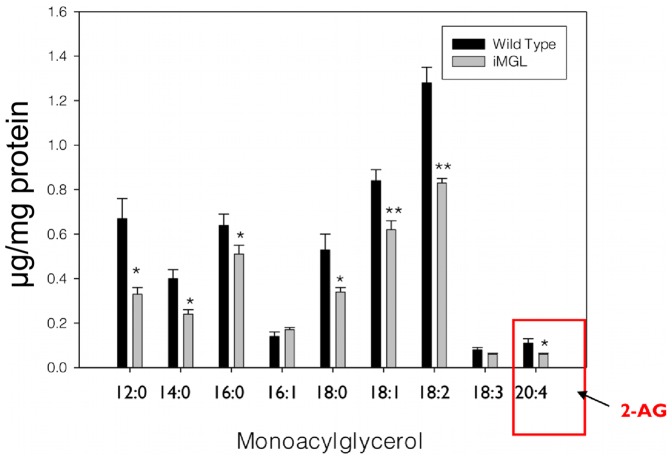
Decreased MG species levels in iMGL mice small intestine by LCMS analysis. (WT; n = 7, iMGL; n = 8). 2-AG, 2-arachidonoyl glycerol. Data represent mean ± S.E. * *p*<0.05, ** *p*<0.01 versus wild type littermates.

Intestinal specific overexpression of murine MGL (mMGL) was driven by the IFABP promoter as illustrated in Fig.S1-A. 12 transgenic founders were initially identified by PCR analysis (Fig.S1-B). Southern blot analyses were performed with genomic DNA from each founder and their offspring to check the copy number and the integrity of transgene integration in the mouse genome (Fig. S1-C). The expected 2 kb bands were detected in the #361, #415, #401, and #359 founders, with extra bands from the 3′ end of the transgene insertion sites into the genome. A less than 2 kb sized band was detected in the #109 line without a 2 kb band, possibly due to a single integration. One of the founders (#361) had multiple insertions of the transgene, indicated by two unpredictable size bands derived from 3′ ends (Fig. S1-C) and further confirmed by two distinct 3′ band patterns in its offspring (F1 generation), so two separate lines (361-1 and 361-2) were established from the #361 founder. The #370 non-transgenic littermate is shown as a negative control for this analysis. Copy numbers varied in the different lines, from a single copy to >50. For most of the subsequent studies, unless otherwise indicated, we used the F1 generation from the #415 founder since 1) it showed a dramatic induction of transgenic MGL mRNA expression (Fig. 1-A), 2) more than 50 copies of intact recombinant mMGL DNA were integrated in a head-to-tail array, and 3) it showed a single insertion pattern, thus avoiding potential problems derived from multiple integrations. Wild type littermates were used as controls.

### Intestinal Specific Overexpression of MGL

Northern blots, probed with a full length mMGL cDNA, demonstrated a robust induction of transgenic MGL mRNA (2 Kb) in the #415, #361-1 and #361-2 lines, whereas no transgenic expression was found in their wild type littermates (Fig. 1-A). Endogenous MGL transcript, corresponding to the 4 kb band, was barely detectable in these adult small intestine samples by Northern analysis, in agreement with our previous findings [13]. The results indicate that overexpression of MGL derived from the IFABP promoter was high compared to endogenous expression levels in the small intestine. Tissue specific expression of the transgene was confirmed by Northern analysis. Transgenic 2 kb mMGL mRNA was found in the small intestine (Fig. 1-B, lane 2) but not in other organs. Brain, adipose, liver, and stomach showed only the 4 Kb endogenous transcript, but no transgenic mRNA (Fig. 1-B), demonstrating intestinal specific expression of the MGL transgene. In agreement with the Northern analysis, a unique SV 40 poly A sequence in the MGL transgene was amplified only in iMGL mice small intestine, and was not detected in other tissues by qPCR (Fig. 1-C). We further examined whether spontaneous changes in endogenous MGL mRNA levels occurred upon transgene expression. For this analysis we amplified the 3′ untranslated region of endogenous MGL mRNA, which is absent in the transgenic MGL transcript. As shown in Figure 1-D, there was no change in endogenous MGL transcript in small intestine. There were also no changes in endogenous MGL expression in liver and brain of iMGL compared to nontransgenic mice, but a small increase was found in adipose tissue (Fig. 1-D). The overexpression used the mouse MGL gene, thus upon translation to protein, endogenous and transgenic enzymes are not distinguishable, only the total amounts of protein and activity are different in the iMGL vs. WT mice. As the IFABP promoter is not regulated by high fat feeding [17], [18], the high fat diet is likely inducing MGL protein expression and activity, as previously described [13]. Figs. 1-E and F show that following 3 weeks of high fat feeding, small intestinal MGL protein and activity levels were significantly higher in the iMGL mice compared to wild type littermates, though the magnitude of induction was not as dramatic as what was found for the iMGL message level (Fig. 1-A and C). The mechanism underlying the blunted overexpression of protein and activity relative to messenger RNA is currently unknown; perhaps putative posttranscriptional controls might be involved, as suggested by our previous studies [13]. Overall, the increased MGL activity in the iMGL animals allows us to examine for the first time the potential function of MGL in the intestine.

### Decreased MG Species Levels in iMGL Small Intestine

In order to confirm a physiological effect of the increased MGL protein and activity in iMGL small intestine, we quantified the levels of individual MG species in iMGL mice small intestine compared to wild type littermates using LC/MS analysis, as described in Methods. The results showed that most of the MG species were significantly lower (20–50%) in iMGL mucosa (Fig.2), in agreement with the reported broad substrate specificity of MGL [19]–[21]. The results clearly demonstrate increased MGL activity after overexpression of the MGL gene in transgenic mice small intestine.

### Normal Metabolic Fates of Dietary Lipids in iMGL Small Intestine

**Figure 3:**
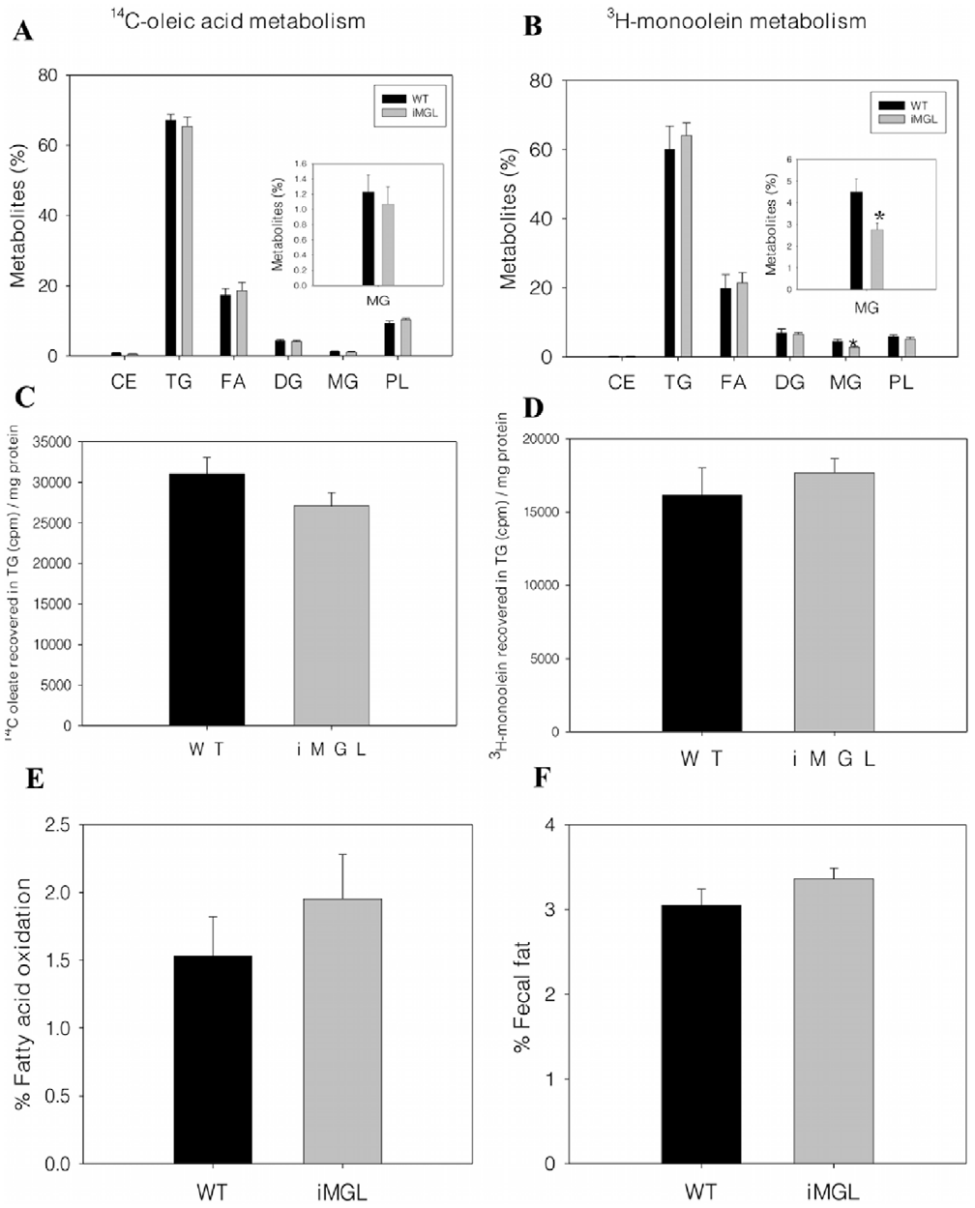
Metabolic fates of dietary fatty acid and monoacylglycerol in iMGL mice small intestine. Metabolism studies were conducted in vivo by administration of radiolabeled lipids into mouse duodenum, and metabolites were assayed as described in Methods. Metabolic fates of (A) dietary ^14^C-oleic acid and (B) dietary ^3^H-monoolein in iMGL mice small intestine. Data are presented as the % of each metabolite relative to total metabolites. Inset shows an enlarged scale for MG (WT; n = 10 (FA) or 5 (MG), iMGL; n = 16 (FA) or 9 (MG). (C) ^14^C oleate incorporation into TG (cpm/mg protein) (WT; n = 10, iMGL; n = 16). (D) ^3^H monoolein incorporation into TG (cpm/mg protein) (WT; n = 5, iMGL; n = 9). (E) Percent fatty acid (^14^C oleate) oxidation in iMGL mice small intestine (WT; n = 10, iMGL; n = 18). (F) Fecal fat analysis. Fecal fat content is presented as the % of stool weight (WT; n = 6, iMGL; n = 5). Data represent mean ± S.E. * *p*<0.05 versus wild type littermates.

FA and *sn*-2-MG are the major hydrolysis products of dietary TG digestion. The metabolic fates of FA and MG were analyzed by intraduodenal introduction of radiolabeled ^14^C-oleic acid or ^3^H-monoolein in vivo. Intestinal FA metabolism in iMGL mice was not altered. In agreement with our previous findings [22], fatty acids were primarily incorporated into TG (70%), with lesser amounts used for phospholipid (PL) synthesis (10%) or other intermediates (Fig. 3-A). For ^3^H-monoolein administration, on the other hand, the percent of radiolabeled ^3^H-MG remaining as MG was almost 40% lower in iMGL mice compared to wild type (wild type; 4.5±0.6% vs. iMGL; 2.8±0.3%, *p*<0.05). No further changes in the acute metabolism of MG were found (Fig. 3-B). The reduced MG levels following ^3^H-monoolein injection are likely due to MGL overexpression in transgenic animals. To determine whether intestinal MGL overexpression affects quantitative TG esterification, the acute (2 min) incorporation of radioactive ^14^C-oleate or ^3^H- monoolein into TG was estimated. No differences in radioactive lipid incorporation per mg of mucosal protein were found (Fig. 3-C and D), suggesting that TG esterification was not affected by MGL overexpression in the small intestine. The expression levels of major lipogenic genes in small intestine were measured by qPCR analysis. There was no compensatory increase in lipogenic gene expression in response to MGL overexpression. In fact, MGAT2 and glycerol-3-phosphate acyltransferase 3 (GPAT3), encoding for the enzymes that initiate TG reesterification in small intestine, were downregulated 40% in iMGL mice (Fig. S2-A). Fatty acid oxidation was also determined following the 2 min intraduodenal administration in vivo, and the percent of ^14^C-oleic acid oxidized to CO_2_ plus acid soluble metabolites was not different in iMGL mice and wild type littermates (Fig. 3-E). To determine whether lipid absorption was altered in iMGL mice, we measured the percent fecal fat content, which was estimated gravimetrically following lipid extraction and based on total fecal weight. The results showed that fecal fat content in iMGL mice was similar to that of the control group, indicating that the efficiency of dietary fat absorption in iMGL mice was not altered (Fig. 3-F).

### Increased Adiposity in iMGL Mice

**Figure 4:**
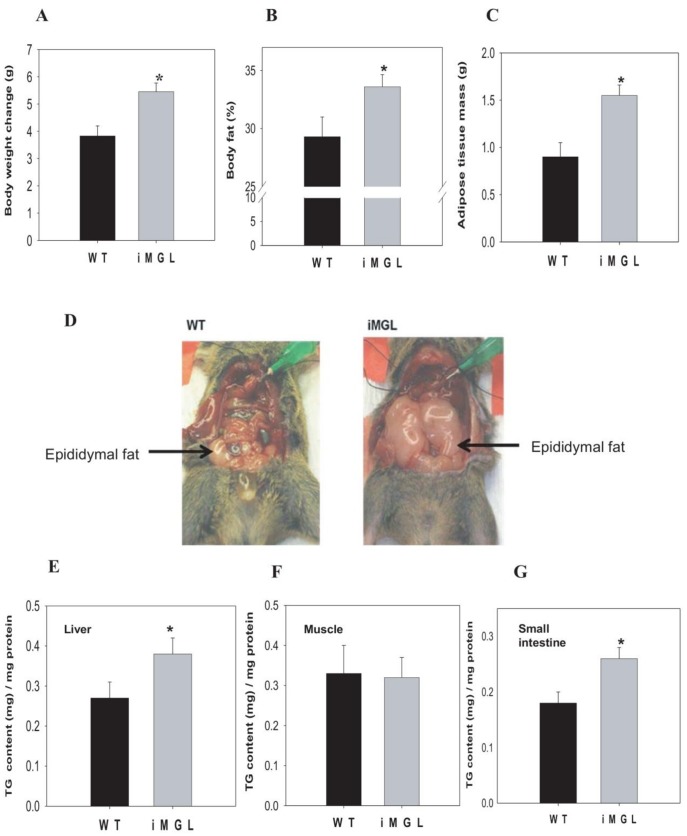
Increased adiposity in iMGL mice after 3 weeks of a high fat (40% kcal) diet. (A) Body weight changes (WT; n = 41, iMGL; n = 57). (B) % body fat determined by DXA measurement (WT; n = 24, iMGL; n = 49). (C) Fat pad weight (total of epididymal and peri-renal depots) (WT; n = 15, iMGL; n = 28). (D) Ventral view of a representative iMGL mouse and a wild type littermate. (E) TG content in liver (WT; n = 6, iMGL; n = 13). (F) TG content in muscle (WT; n = 8, iMGL; n = 16). (G) TG content in intestinal mucosa (WT; n = 9, iMGL; n = 14). Data represent mean ± S.E. * *p*<0.05 versus wild type littermates.

**Figure 5:**
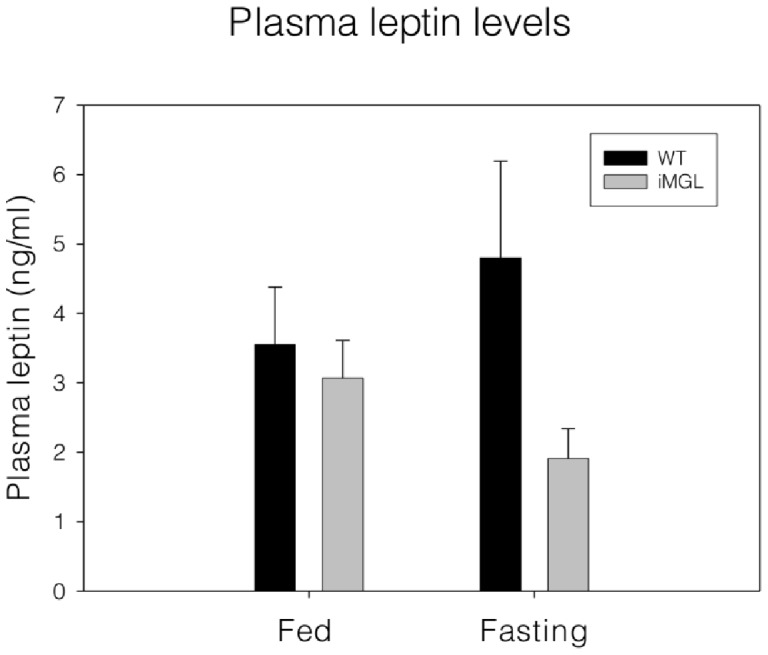
Leptin levels in iMGL mice plasma. Plasma leptin levels (n = 13 per group). Data represent mean ± S.E.

iMGL animals were viable and healthy. No abnormalities or distinguishable phenotypes were observed during growth. At 3 months of age, there was no significant difference in body weights, although the iMGL mice showed a trend toward higher body weight (wild type; 29.1±0.5g vs. iMGL; 30.4±0.5g, *p* = 0.07). After 3 weeks of high fat feeding, however, weight gains in the iMGL mice were significantly higher than those of wild type littermates (Fig. 4-A). Another transgenic line (#361-1 line) also showed a similar result, with higher body weight gain relative to wild type littermates (Fig. S3). The percent body fat was significantly higher and adipose tissue mass was double in iMGL mice, with no difference in lean body mass (wild type; 23.1±0.4g vs. iMGL; 22.9±0.4g) compared to wild type mice following 3 weeks of high fat feeding (Fig. 4-B and C). Ventral views of iMGL mice and their littermate controls also clearly demonstrated the increased adiposity in iMGL mice; representative images are shown in Fig. 4-D. Ectopic fat deposition was found in liver, with TG content significantly elevated (20%) (Fig. 4-E), but muscle TG level was not different (Fig. 4-F). Small intestinal mucosa also had increased TG content (Fig. 4-G). Lipid metabolic gene expression in iMGL mice was examined in liver and adipose tissues by qPCR analysis. No substantial changes in hepatic gene expression were found (Fig. S2-B), however in adipose, there was a significant upregulation of leptin, PPARγ, MGAT, and DGAT2 whereas lipolytic ATGL transcript was reduced (Fig. S2-C). These alterations in adipose tissue gene expression are generally consistent with the increased adiposity of the iMGL mice relative to WT littermates, although an upregulation of the expression of oxidative CPT1 and ACOX1 transcripts were also noted. Plasma TG levels in iMGL mice were significantly higher in the fed state, but not in the fasting condition (Table S1). In addition, although fasting blood glucose levels were not altered, there was a trend toward increased plasma insulin levels in the transgenic mice compared to those of littermate controls (Table S1). The absence of significant changes in fasting compared to fed insulin and leptin levels was unexpected; a blunted metabolic response to fasting in obese mice has been reported by Ueno et al. [23], thus it is possible that the high fat feeding in the present study may have resulted in a lack of change in these parameters. Surprisingly, however, despite the markedly increased adiposity of iMGL mice, which is consistent with the significant increase in adipose leptin transcript (Fig.4 and Fig. S2-C), plasma leptin levels were not proportionally higher in the transgenic animals. In fact, fasting leptin levels were 2.5-fold lower in the transgenic animals, although this observation did not reach statistical significance (Fig.5, *p* = 0.07).

### Increased Food Intake and Decreased Energy Expenditure in iMGL Mice

**Figure 6:**
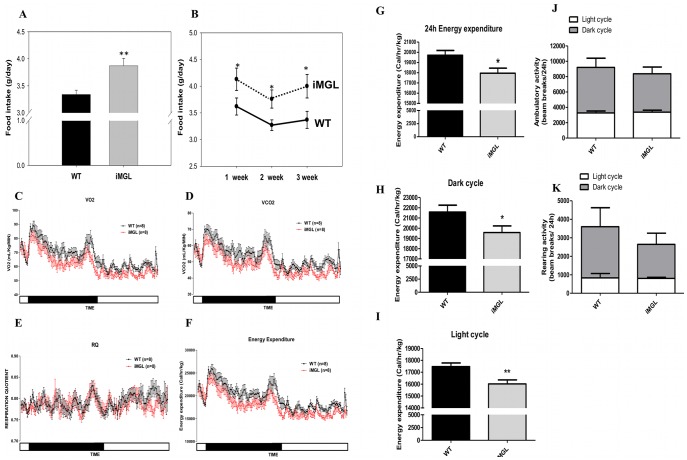
Altered energy intake and expenditure in iMGL mice. (A) Average daily food intake per mouse over the 3 week high fat feeding trial (WT; n = 15, iMGL; n = 20). (B) Time course of average daily food intake per mouse for each week (WT; n = 15, iMGL; n = 15). (C) Time course of oxygen consumption during 24 hr. Values are expressed as ml/kg/min (WT; n = 8, iMGL; n = 8). Note that two-way ANOVA analysis showed a significant difference in genotype (*p*<0.05 versus WT). (D) Time course of CO_2_ production during 24 hr. Values are expressed as ml/kg/min (WT; n = 8, iMGL; n = 8). Two-way ANOVA showed a significant difference in genotype (*p*<0.05 versus WT). (E) Time course of respiratory quotient (RQ) values (WT; n = 8, iMGL; n = 8). (F) Time course of energy expenditure during 24 hr. Values were expressed as calorie/hour/kg body weight (WT; n = 8, iMGL; n = 8). Two-way ANOVA showed a significant difference in genotype (*p*<0.05 versus WT). On the X axis, the black bar represents the dark period and the white bar is the light period. (G) Average energy expenditure over 24 h. (H) Average energy expenditure in the dark period. (I) Average energy expenditure in the light period. All indirect calorimetry measurements were collected every minute for 24 hr. (J) Ambulatory activity and (K) Rearing activity in iMGL mice (WT; n = 6, iMGL; n = 6). Gray bars represent activities from dark cycle and white bars for light cycle. Data represent mean ± S.E. *p≤0.05, ** *p*<0.01 versus wild type littermates.

Cumulative food intakes during the high fat feeding trial were measured and average daily food intake per mouse was calculated. A significant 15% increase in the food intake of iMGL mice was found compared to littermate controls (Fig. 6-A), indicating that the iMGL mice were hyperphagic. This hyperphagic behavior was observed in the first week of feeding, prior to substantial differences in weight gain, and was sustained throughout the entire feeding period (Fig. 6-B). Metabolic rates of iMGL and wild type mice were monitored for 24 hr. by indirect calorimetry, with results demonstrating significant decreases in both oxygen consumption (VO_2_) and carbon dioxide production (VCO_2_) over the entire time course, both in the dark phase and the light phase (Fig. 6-C, and D). Thus, respiratory quotient (RQ) values in both genotypes were similar (WT 0.78±0.01/iMGL; 0.78±0.01), indicating no alteration in fuel selection in iMGL mice (Fig. 6-E). Expected circadian RQ fluctuations were not observed in both genotypes (Fig. 6-E), another example of a possible blunted metabolic response upon high fat diet feeding, as described by Satoh et al. [24]. Interestingly, despite the fact that lean body mass was not changed, energy expenditure in iMGL mice, calculated using the Weir formula [25], was lower at all time points (Fig. 6-F). The calculated average metabolic rate during the 24 hr measurement was reduced by 9% in iMGL mice (p<0.05), with significant reductions in both the dark (p = 0.05) and light (p<0.01) phases (Figs. 6-G-I). To further address the origin of the decreased energy expenditure, physical activity was measured using beam-break instrumentation. The results show a trend, albeit not statistically significant, toward decreased activity, particularly rearing movement, in the iMGL compared to wild type littermates in the dark phase (Fig. 6-J, 6-K). Overall, these observations suggest that both increased food intake and decreased metabolic rate contribute to the increased body weight and adiposity in iMGL mice.

### Appetite Signaling in iMGL Mice

**Figure 7:**
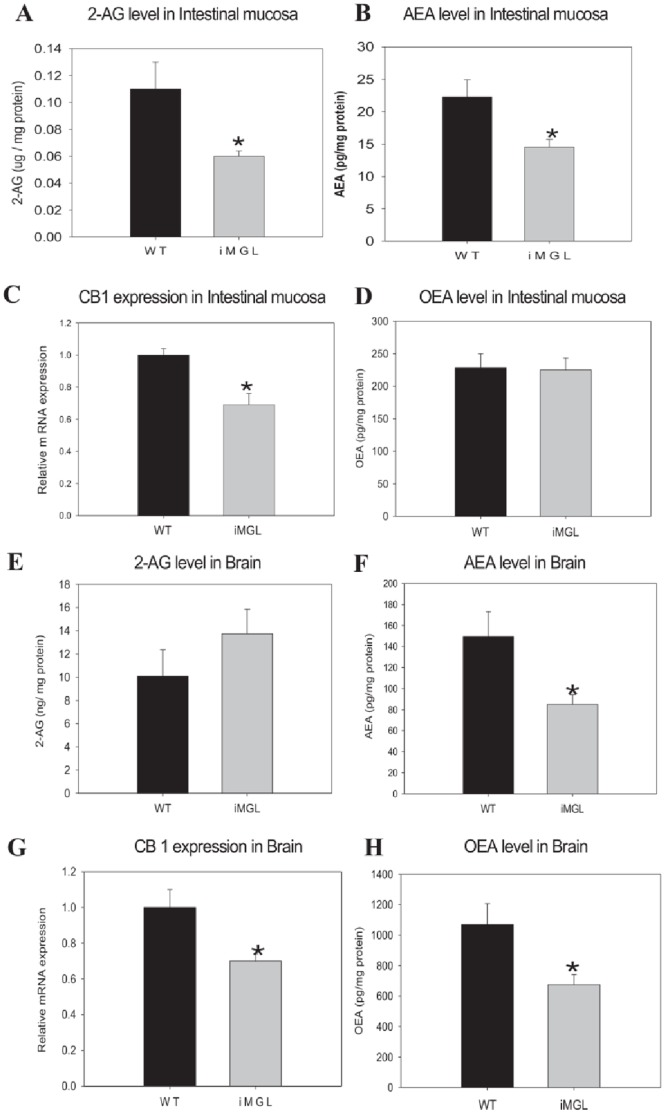
Endocannabinoid (EC) levels and CB1 expression in iMGL mice small intestine and brain. (A) 2-AG level in iMGL mice small intestine (n = 6 per group). (B) AEA level in iMGL mice small intestine (n = 6 per group). (C) CB1 mRNA expression in iMGL mice small intestine by QPCR analysis (n = 6 per group). Values are presented relative to the expression of the wild type littermates set to 1. (D) OEA levels in small intestinal mucosa (n = 8per group). (E) 2-AG level in iMGL mice brain (WT; n = 5, iMGL; n = 9). (F) AEA level in iMGL mice brain (WT; n = 5, iMGL; n = 9). (G) CB1 mRNA expression in iMGL mice brain by QPCR analysis (n = 6 per group). Values are presented relative to the expression of the wild type littermates set to 1. (H) OEA levels in brain (WT; n = 5, iMGL; n = 9). Data represent mean ± S.E. * *p*<0.05 versus wild type littermates.

To examine the mechanism underlying the hyperphagic behavior observed in iMGL mice, we measured endocannabinoid levels and CB1 receptor expression both in small intestine and brain, as well as levels of the anorexic lipid mediator, oleoylethanolamide (OEA). As mentioned above, 2-AG levels were significantly lower in iMGL intestine (Fig. 3 and 7-A). Anandamide (AEA) levels were also significantly lower in intestinal mucosa compared to those found in wild type littermates (Fig.7-B), as was CB1 receptor expression (Fig. 7-C). OEA levels were unchanged (Fig. 7-D). In brain, the iMGL mice showed a trend toward increased 2-AG levels (Fig.7-E); lower levels of anandamide and lower CB1 expression were found in brain, as in intestine (Fig 7-F and G). Brain OEA levels were significantly decreased (Fig. 7-H). Expression of the brain neuropeptides neuropeptide Y (NPY), agouti related protein (AGRP), pro-opiomelanocortin (POMC) and cocaine and amphetamine regulated transcript (CART) were also measured by qPCR. No significant changes were found except for a reduction in NPY (Fig.S4). Fasting plasma levels of 2-AG, AEA, ghrelin, and N-acylphosphatidylethanolamines (NAPEs) showed a trend to be decreased in iMGL mice, and plasma PYY was not changed (Table S1).

## Discussion

Overexpression of MGL in proximal intestinal mucosa, which resulted in lower levels of almost all acyl chain species of MG including the endocannabinoid 2-AG, led to profound systemic changes in energy homeostasis. After only 3 weeks on a relatively moderate 40% kcal high fat diet, the absolute increases in fat mass in the iMGL mouse were comparable in degree to reciprocal fat mass losses found in the DGAT1 and MGAT2 KO mice [26], [27]. The iMGL mice also displayed increased TG accumulation in adipose tissue and liver. An obese phenotype was unexpected, as we had hypothesized that MGL overexpression in mucosa would lead to less TG synthesis via the MGAT pathway. Instead, we found that the acute intestinal metabolism of MG and FA were not substantially altered, and intestinal TG synthesis was intact. In fact, there was a significant increase in mucosal TG accumulation, suggesting the deposition of ectopic lipid in the intestine as well as in the liver. Studies are underway to determine whether the mucosal steatosis in the iMGL mouse may, in part, be due to impaired intestinal lipid secretion. We have recently shown that the genetically obese ob/ob mouse, as well as high fat-fed wild type C57BL6 mice, show increased intestinal mucosal TG levels relative to low fat-fed mice, thus the increased mucosal TG in iMGL mice may be secondary to the obese phenotype [28].

The profound systemic effects of intestinal MGL overexpression point to an important link between intestinal lipid metabolism and whole body energy balance. Interestingly, similar whole body effects were observed in mice null for either of the two key enzymes in the intestinal TG esterification pathway, DGAT1 and MGAT2, where, as in the present results, the knockout animals displayed more substantial changes in whole body energy homeostasis than in intestinal lipid metabolism itself [26], [27], [29]. Both these null models showed an increased metabolic rate, resulting in lean phenotypes [26], [27], [29]. The DGAT1 KO mice displayed an increased food intake despite the lean phenotype, and the hyperphagia became more profound when animals were exposed to cold; the mechanisms underlying these changes remain unknown [29]. In addition to the gain and loss of function studies in mice, an association between MG metabolism and obesity has been reported in rats and humans as well [30], [31]. In Otsuka Long-Evans Tokushima Fatty rats, a model of spontaneous type 2 diabetes, a robust induction in intestinal MGAT activity has been reported, implicating a contribution of intestinal MG metabolism to metabolic perturbation [30]. In addition, linkage and genome-wide association analyses of obesity-related phenotypes identified MGAT1 as a candidate gene associated with body weight [31]. These associations may not arise directly through the enzymatic pathways themselves, but possibly via modulating signaling pathways which regulate appetite and energy expenditure. Indeed, Shi and Cheng have discussed a potential interaction of EC signaling with MGAT and DGAT activities, since 2-AG is both a signaling molecule through the EC system and a substrate not only of MGAT but also of DGAT enzymes [32].

In recent decades, knowledge of GI endocrine functions has expanded rapidly, along with the discovery of many gut satiety signals, including the anorexic lipid mediators, OEA and NAPE [33]–[35]. Biosynthesis of both lipid metabolites is stimulated in enterocytes in response to dietary fat ingestion, and food-derived oleic acid is known to be a primary precursor for OEA synthesis [35]. Long chain fatty acyl coA (LCFAcoA) derived from dietary fat has also been reported to participate in GI lipid sensing, reducing food intake by inducing CCK release [36]. In addition to their anorexic effects, intestinal lipid messengers also regulate energy metabolism in other peripheral tissues. For example, OEA stimulates lipolysis in adipose tissue and increases FA oxidation in muscle and liver via PPAR-α activation [37]. Further, Wang et al, recently showed that proximal intestinal LCFAcoA remotely controls hepatic glucose production via an intestine-brain-liver neural connection [38]. Taken together, intestinal lipids are emerging as critical metabolic signaling molecules in the control of whole body energy balance. The intestinal epithelium is exposed to high levels of 2-monoacylglycerols during dietary lipid digestion and absorption, and these lipid metabolites, including 2-AG and perhaps other monoacylglycerol species, are also likely involved in appetite regulation, as we demonstrate in the present study. As with the effects of DGAT and MGAT gene ablations on whole body energy balance [26], [27], the precise mechanisms remain to be elucidated.

The actions of intestinal MGL and intestinal 2-AG on food intake and eating behavior via the EC system have not been as intensively investigated as they have in the central nervous system. Functional analysis of the gut EC system has emphasized gastroprotection against inflammation and the regulation of intestinal motility [39], [40]. However, recent evidence suggests that the gut EC system may also be involved in the control of food intake [41]–[45]. Significant increases in intestinal anandamide (AEA) and 2-AG levels were reported during food deprivation, and re-feeding reversed these effects [41], [42]. CB1 expression in the vagus nerve termini innervating the gastrointestinal tract was regulated by feeding status as well [43]. Dysregulation of gut EC signaling was reported in Zucker (*fa/fa*) rats; basal intestinal AEA and 2-AG levels were elevated 5–10 fold and the postprandial reduction in small intestine EC levels was blunted compared to lean animals [44]. Further, intraduodenal injection of the CB1 receptor antagonist rimonabant, potently inhibits dietary fat intake, suggesting a direct action on CB1 receptors in the gut, rather than central mediation [45]. Together, these results point to a potential role for intestinal EC in regulating food intake and energy balance. In agreement with these observations, here we show that intestinal MGL levels significantly altered both local and central EC systems, resulting in a dysregulation in food intake as well as an alteration in metabolic rate.

Hyperactivity of the central EC system results in hyperphagic behavior and increased adiposity, and MGL works to terminate EC signaling by breaking down MG, an endogenous substrate for CB receptors [46]. In the case of the intestine-specific overexpression of MGL, we have a paradox in that MGL induction in the small intestine caused a phenotype associated with central EC system activation rather than termination. Interestingly, Taschler et.al reported that global MGL knockout mice exhibit neither the expected hyperphagic behavior nor an obese phenotype when fed a very high fat diet, despite a robust increase in 2-AG levels in brain [47]. Another whole body MGL knockout mouse, where animals were fed a chow diet, actually showed a decrease in body weight, and none of the expected alterations in neuropathic and inflammatory pain sensitivity [48]. Both groups propose CB1 receptor desensitization as a plausible explanation for the absence of cannabinoid-like effects in MGL KO [47], [48]. It is a noteworthy that peripheral MGL has other important functions in addition to EC signaling. In adipose, MGL catalyzes the final step of TG hydrolysis [49]. Liver also expresses a considerable amount of MGL [50], but the precise function of hepatic MGL is unknown. Thus, the unexpected phenotype of global MGL KO mice may, perhaps, originate from its diverse function in different tissues. Nevertheless, in a line with the desensitization hypothesis, it is possible that chronic inhibition of the EC system by MGL overexpression may result in a compensatory upregulation of other signaling pathways.

To begin to understand the underlying mechanism of the iMGL phenotype, we determined the levels of several other known appetitive signals in brain and plasma. In agreement with the markedly increased adiposity of iMGL mice, we detected a significant increase in adipose leptin mRNA expression in iMGL mice (Fig.S2-C). Nevertheless, the circulating leptin levels were unchanged and likely lower in iMGL mice than in their wild type littermates (Fig.5), since the average leptin levels in iMGL were almost 3-fold lower than in controls but due to the large variability among mice the decrease did not reach statistical significance (*p* = 0.07). An uncoupling between plasma leptin levels and adiposity was also found in the CD36 null mouse as well as in obese human subjects with a common IRS-1 polymorphism [51], [52]. In addition, uncoupling between adipose leptin mRNA levels and plasma levels has also been reported, along with a number of posttranscriptional mechanisms regulating leptin secretion [53]–[55]. The mechanisms underlying the discordant regulation of leptin in iMGL mice is currently unknown, but could be related to secretion from the fat cells, or to plasma leptin degradation. The lack of change or, perhaps, decrease, in circulating leptin levels observed in iMGL mice may contribute to their hyperphagic behavior. It is also of interest that circulating plasma NAPE levels tend to be reduced in iMGL mice (Table S1). NAPE is believed to be a dietary fat-driven satiety factor; it is synthesized in small intestine and released into the plasma in response to fat ingestion, inhibiting food intake [34]. Thus, reduced plasma NAPE levels may also contribute to iMGL hyperphagia.

In summary, we found a clear perturbation of whole body energy metabolism in mice overexpressing MGL specifically in the small intestine. Increased adiposity secondary to hyperphagia and hypometabolic rate in iMGL mice suggests that intestinal MGL may be envisioned as a new metabolic regulator involved in the control of whole body energy homeostasis. Detailed molecular mechanisms remain to be elucidated; such studies will likely lead to another avenue for understanding intestinal MG, in addition to its role as a metabolic intermediate in dietary lipid assimilation.

## Methods

### Ethics Statement

All animal work was approved by the Rutgers University Laboratory Animal Services animal care review committee under approval number 92-052.

### Generation of Transgenic Mice

Intestinal specific overexpression of mMGL was driven by the IFABP promoter (region -1178 to +28 nucleotides of the rat IFABP gene) [56], generously provided by Dr. Jeffry Gordon (Washington U., St. Louis MO). As diagrammed in Fig.S1-A, the mouse MGL coding region and SV40 poly A and intron sequences were subcloned into the vector containing the IFABP promoter. The linearized transgene was purified and injected into mouse (hybrid strain, C57/BL6J X SJL) pronuclei and then transplanted into surrogate females to produce potential transgenic founders. Transgenesis was done by the transgenic core facility at U. of Michigan. Initial transgene detection among the potential founders was done by PCR analysis of genomic DNA samples from tail biopsies. Primers were designed so that the PCR reaction produces a unique 400 bp junction fragment spanning the middle of the MGL coding region and part of the SV40 poly A sequence in transgenic animals: forward 5′-GAG TCA GGA CAA AAC ACT CAA GAT GTA -3′, reverse 5′-ACT AGA TGG CAT TTC TTC TGA GCA AAA C-3′ (Fig.S1-B). Southern blot analysis was performed in order to verify the number of copies and the integrity of the transgene in the mouse genome. 25 µg of genomic DNA was digested with BamH1, separated, and transferred onto nylon membranes (GE Healthcare, Piscataway, NJ). Integrated recombinant DNA was detected by [^32^P] labeled probes for partial sequences of a) SV40 poly A signal, b) IFABP promoter region, or c) the junction region between the MGL coding region and SV 40 poly A sequences (Fig. S1-C).

### Animals, Diets, and Tissue Collection

Animals were maintained on a 12 hr light and dark cycle and fed a regular chow diet (Purina Mouse Chow 5015, Purina Co., St. Louis, MO) ad libitum after weaning. Male mice aged ∼ 3 months old were housed two per cage, and fed with purified rodent diet consisting of 40% fat by calories (D12327, Research Diets, New Brunswick, NJ), for 3 weeks ad libitum. Food intakes and body weights were monitored weekly and body composition was measured using the Piximus (Perkin Elmer Life Science, Boston, MA) mouse DXA instrument at the end of the feeding period. Animals were anesthetized prior to either metabolism studies or tissue collection. Unless specified, experiments were done in the fed state. For fasting, food was withdrawn for 16–18 h overnight. All tissues were immediately frozen in ethanol/dry ice and kept at −70°C.

### Northern and Western Blot Analysis for Transgenic MGL Expression and in vitro MGL Assay

Transgenic MGL mRNA and protein expression were analyzed by Northern and Western blot and MGL activity was determined by measuring the release of fatty acid from radiolabeled *sn*-2-MG as described previously [13].

### In vivo Fatty Acid and sn-2-MG Metabolism

The anabolic and catabolic metabolic fates of dietary oleic acid and *sn*-2-monoolein in small intestine were determined in vivo using intra-duodenal radiolabeled lipid administration as described [22]. Mice were anesthetized with a mixture of ketamine/xylazine/promazine (80/100/150 mg/kg, respectively) via intraperitoneal injection prior to lipid administration. Less than 40 nmol of each labeled lipid, either [^3^H] sn-2-monoolein or [1-^14^C] oleic acid, was administrated via cannula to the mouse duodenum as a mixture with 10 mM sodium taurocholate. After 2 min, mice were sacrificed, and the entire small intestine from pylorus to cecum was immediately excised. The intestine was rinsed twice with saline, and the mucosa was harvested by scraping. Tissue lipids were extracted using the Folch method [57]. Lipid extracts were spotted on standard silica gel plates (Sigma, St. Louis, MO) and separated by a solvent system of hexane/ethyl ether/acetic acid, 70∶30∶1, v/v. Quantification of each lipid species was analyzed using ImageQuaNT software (Molecular Dynamics, Sunnyvale, CA). For [3H] sn-2- monoolein metabolism, TLC plates were scraped and counted using a scintillation counter (Perkin Elmer Life Science, Boston, MA).

### Tissue TG

To determine tissue TG content, intestinal mucosa, liver, and muscle lipid extracts [57] were spotted onto TLC plates with lipid standards, separated, and visualized by iodine staining. Quantification was performed using Image J software (NIH).

### Fecal Fat Analysis

Fecal fat content was estimated gravimetrically as described by Newberry et al. with minor modification [58]. Briefly, 0.5 g feces were homogenized in 5 ml of chloroform/methanol (2∶1) solution. Lipid was extracted with an additional 15 ml of chloroform/methanol (2∶1). Fecal fat weight was measured in pre-weighed glass vials. Percent fecal fat was calculated based on total fecal weight.

### Fatty Acid Oxidation

Intestinal FA oxidation was measured as described previously [22]. Total oxidation was calculated as the amount of perchloric acid- soluble radioactivity plus ^14^CO_2_ captured on filter papers soaked in benzethonium hydroxide.

### Blood Chemistry

Plasma TG, free fatty acid, and total cholesterol levels were determined using colorimetric assay kits (Wako, Richmond, VA). Fasting blood glucose levels were measured using the Accu-Chek (Roche Diagnostics, Indianapolis, IN) glucose meter from mouse tail snips. Insulin, leptin, active ghrelin, and total PYY levels were analyzed using a Millipore Elisa Kit at the Mouse Metabolic Phenotyping Center at the University of Cincinnati (DK59630).

### LCMS Analysis

Individual MG species, anandamide (AEA), oleoylethanolamide (OEA), and NAPE levels in small intestinal mucosa, brain, and plasma were estimated by LC/MS analysis using a Dionex UltiMate 3000 HPLC (Dionex, Sunnyvale, CA, USA) coupled to an AB SCIEX 4000 QTRAP® mass spectrometer (AB SCIEX, Foster City, CA, USA). The solvent gradient was modified from Homan and Anderson [59]. The peaks corresponding to each analyte were integrated and concentrations were calculated using Analyst 1.4.2 software in Windows XP. Tissue lipids were extracted [57], dried down, and reconstituted in isooctane: tetrahydrofuran (9∶1, v/v) for injection. For MG analysis, ionization was carried out with the PhotoSpray® atmospheric pressure photoionization source operated in positive ion mode. A standard curve containing 50 pg to 10 ng of each of 9 monoacylglycerols (including 2-AG) was run prior to and immediately after the sample lipid extracts. Lipid extract from 8 µg and 900 µg of tissue protein were used for intestinal and brain MG quantification respectively. For plasma samples, an amount of extract equivalent to 32–60 µl plasma was injected. For AEA and OEA the standard curves included both compounds at concentrations ranging from 2.5 pg to 10 ng per injection. Lipid extract from 400 µg and 600 µg of tissue protein were used for intestinal and brain ethanolamide quantification respectively. For plasma, the OEA/AEA and NAPE methods were combined so that both compound classes could be analyzed in the same injection. NAPEs were analyzed using three parallel precursor ion scans for the three head groups determined (N-palmitate, oleate, and arachidonate acyl groups). Extract equivalent to 90–220 µl plasma was injected.

### Energy Expenditure Measurements

Mouse metabolic rates were measured by indirect calorimetry, using the PhysioScan instrument (AccuScan Instruments Inc.) at the Mouse Metabolic Phenotyping Center at the University of Cincinnati (DK 59630). Energy expenditure was calculated as described by Weir with minor modification [25].

### Physical Activities Measurements

Physical activity was measured using beam-break instrumentation. Mice (415 line backcrossed to SJL, F10 generation) were placed in single housing metabolic chambers of the Oxymax Instrument (Columbus Instruments, Columbus, OH). All mice had ad libitum access to food (40% HFD) and water. After 24 hours of acclimatization, activity of each mouse was monitored continuously for 24 hours by infrared beams that cover horizontal and vertical axes. Total number of beam breaks (for total activity, ambulatory activity, and rearing activity) were recorded in 10 minute bins and combined for the light phase (7 am - 7 pm), dark phase (7 pm - 7 am), and total 24 hour period.

### Quantitative RT-PCR

Preparation of RNA samples and QPCR reactions were performed as described previously [13]. Primer sequences were retrieved from Primer Bank (Harvard Medical School QPCR primer database), and are listed in Table S2. QPCR reactions were performed in triplicate using an Applied Biosystems 7300 instrument. Each reaction contained 80 ng of cDNA, 250 nM of each primer and 12.5 ul of SYBR green master mix (Applied Biosystems, Foster City, CA) in a total volume of 25 ul. Relative quantification of expression was calculated using the comparative Ct method normalized to β-actin.

## Supporting Information

Figure S1 Generation of transgenic mice (iMGL) overexpressing MGL in small intestine. (A) IFABP promoter/MGL recombinant vector. (B) PCR screening for transgenic animals (A representative picture). A unique 400 bp sequence only present in transgenic animals was the target for amplification. Bottom gel shows β-globin amplification, used for checking the integrity of the genomic DNA samples. Numbers on the bottom indicate individual potential founders. (C) DNA analysis of various transgenic founders by Southern blotting. Top diagram describes the design of a Southern analysis. Partial sequence of 0.8 kb region (thick bars) was used as a probe to detect a 2 kb sequence (thin bar) present in the transgene. Bottom blot shows the expected 2 kb band with various copy numbers in several transgenic lines along with their extra bands (unpredictable sizes from 3′end insertion into the mouse genome). Right side of blot shows copy number standard from 0 to 100.
(TIF)

Figure S2 Lipid metabolic gene expression in iMGL mice by qPCR analysis. (A) Lipogenic gene expression in small intestine (n = 6 per group). **(**B) Hepatic gene expression (n = 7 per group). (C) Adipose tissue gene expression (n = 8 per group). Values are presented relative to expression of the wild type littermates. Data represent mean ± S.E. * *p*<0.05, ** *p*<0.01 versus wild type littermates.
(TIF)

Figure S3 Body weight changes in 361-1 line iMGL mice after 3 weeks of a high fat (40% kcal) diet. F4 generation mice backcrossed with SJL (WT; n = 3, iMGL; n = 4). Data represent mean ± S.E. ** *p*<0.01 versus wild type littermates.
(TIF)

Figure S4 Brain neuropeptide mRNA expression in iMGL mice by QPCR analysis. (A) Neuropeptide Y (NPY), (B) Agouti-related protein (AGRP), (C) Ghrelin receptor (D) Pro-opiomelanocortin (POMC), (E) Cocaine and amphetamine regulated transcript (CART), and (F) Leptin receptor. Values are presented relative to the expression of the wild type littermates set to 1. Data represent mean ± S.E. * *p*<0.05 versus wild type littermates (n = 6 per group).
(TIF)

Table S1 Plasma parameters in iMGL mice. Plasma TG, cholesterol, fatty acid, 2-AG, glucose and insulin levels (n = 8∼16), gut peptides (active ghrelin and total PYY) (n = 4), and AEA, OEA, and NAPEs (2 pooled samples from total of n = 8 mice per group). Data represent mean ± S.E. * *p*<0.05 versus wild type littermates.
(DOC)

Table S2 QPCR primer sequences.
(DOC)
