# Supplementary figures and images for: Over-Expression of Monoacylglycerol Lipase (MGL) in Small Intestine Alters Endocannabinoid Levels and Whole Body Energy Balance, Resulting in Obesity
Source: PLoS One. 2012 Aug 28;7(8):e43962. doi: 10.1371/journal.pone.0043962 (PMC3429419; doi:10.1371/journal.pone.0043962)

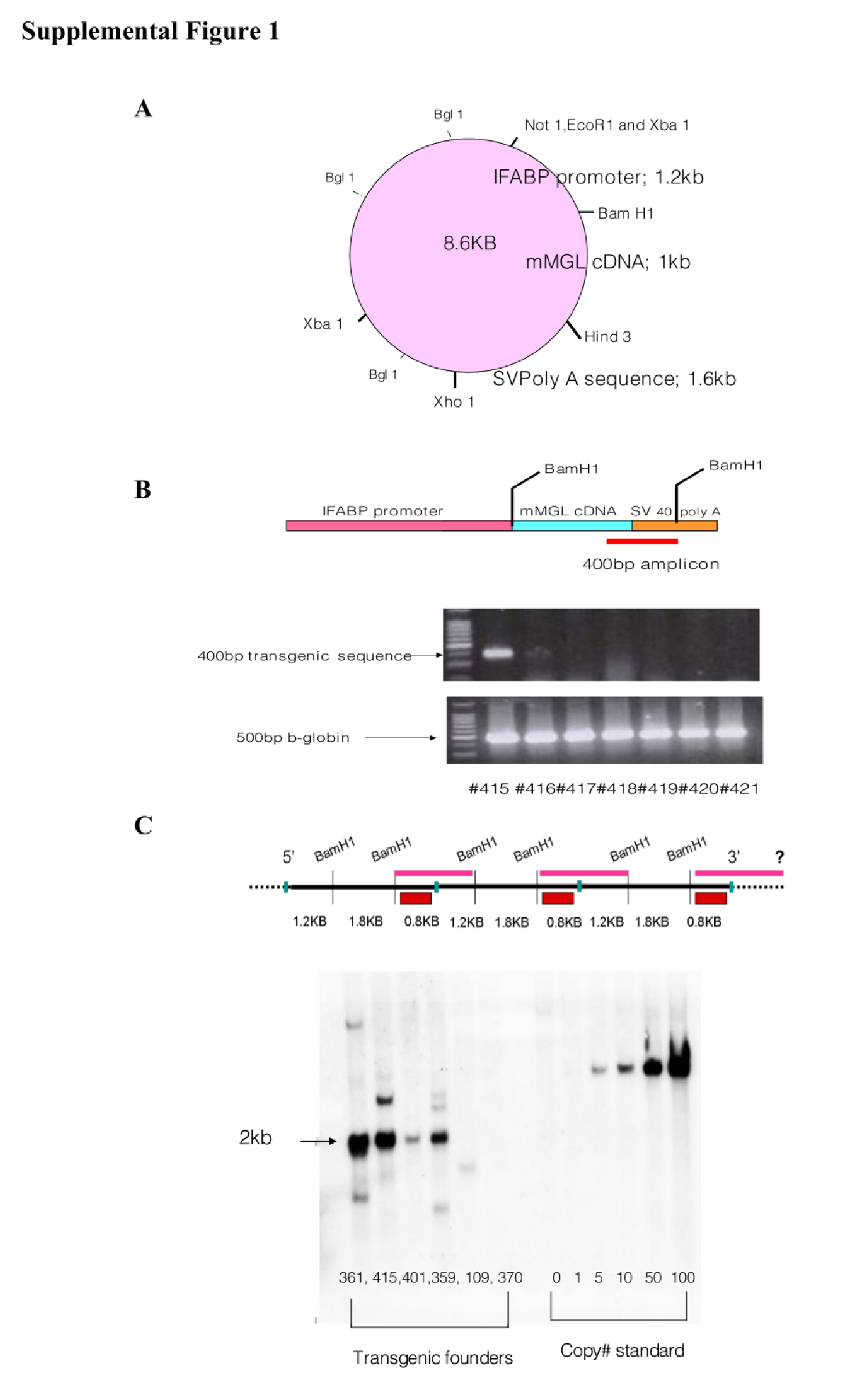

Supplement: Figure S1 — Generation of transgenic mice (iMGL) overexpressing MGL in small intestine. (A) IFABP promoter/MGL recombinant vector. (B) PCR screening for transgenic animals (A representative picture). A unique 400 bp sequence only present in transgenic animals was the target for amplification. Bottom gel shows β-globin amplification, used for checking the integrity of the genomic DNA samples. Numbers on the bottom indicate individual potential founders. (C) DNA analysis of various transgenic founders by Southern blotting. Top diagram describes the design of a Southern analysis. Partial sequence of 0.8 kb region (thick bars) was used as a probe to detect a 2 kb sequence (thin bar) present in the transgene. Bottom blot shows the expected 2 kb band with various copy numbers in several transgenic lines along with their extra bands (unpredictable sizes from 3′end insertion into the mouse genome). Right side of blot shows copy number standard from 0 to 100. (TIF) [file pone.0043962.s001.tif]

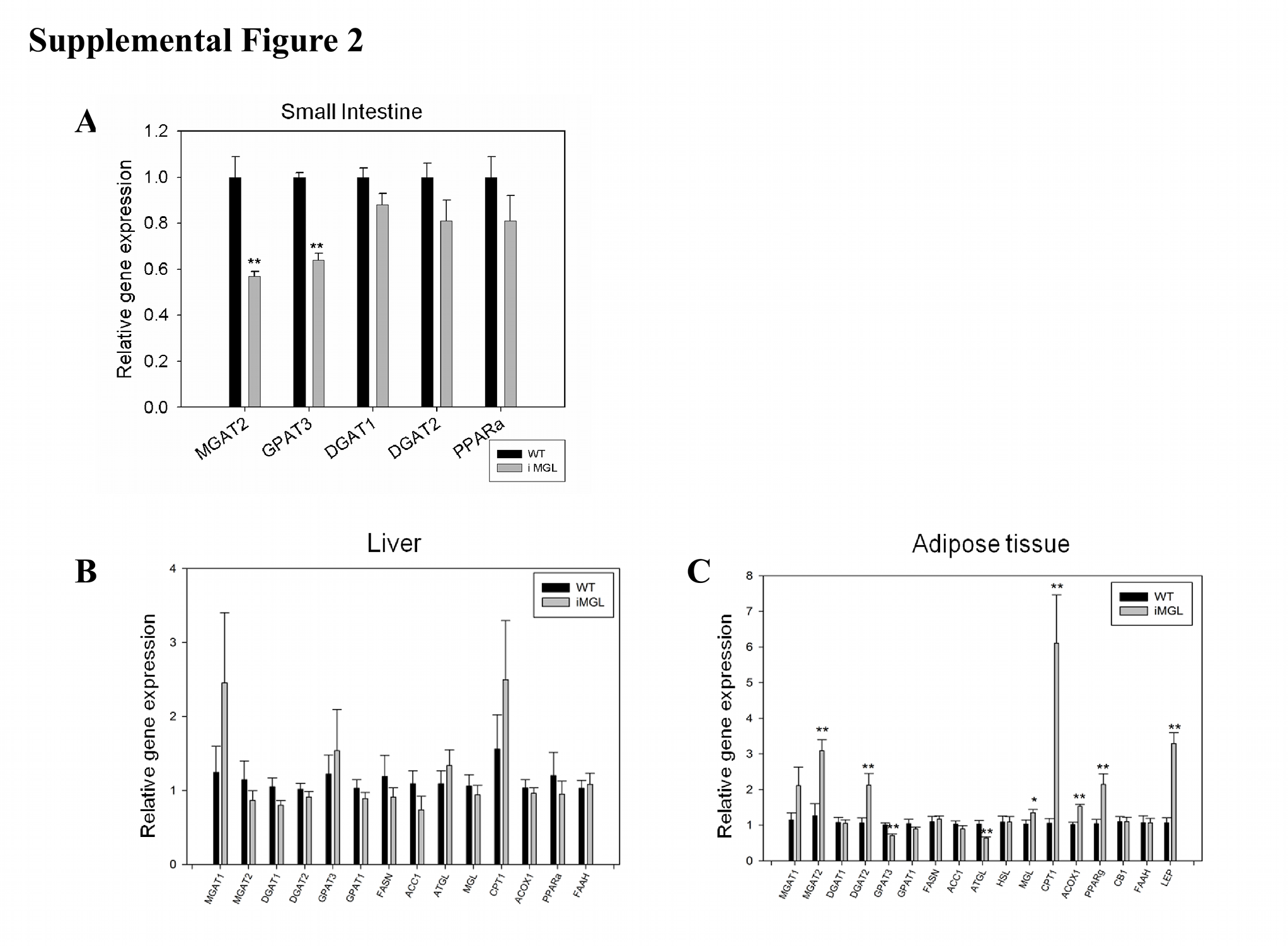

Supplement: Figure S2 — Lipid metabolic gene expression in iMGL mice by qPCR analysis. (A) Lipogenic gene expression in small intestine (n = 6 per group). (B) Hepatic gene expression (n = 7 per group). (C) Adipose tissue gene expression (n = 8 per group). Values are presented relative to expression of the wild type littermates. Data represent mean ± S.E. * p<0.05, ** p<0.01 versus wild type littermates. (TIF) [file pone.0043962.s002.tif]

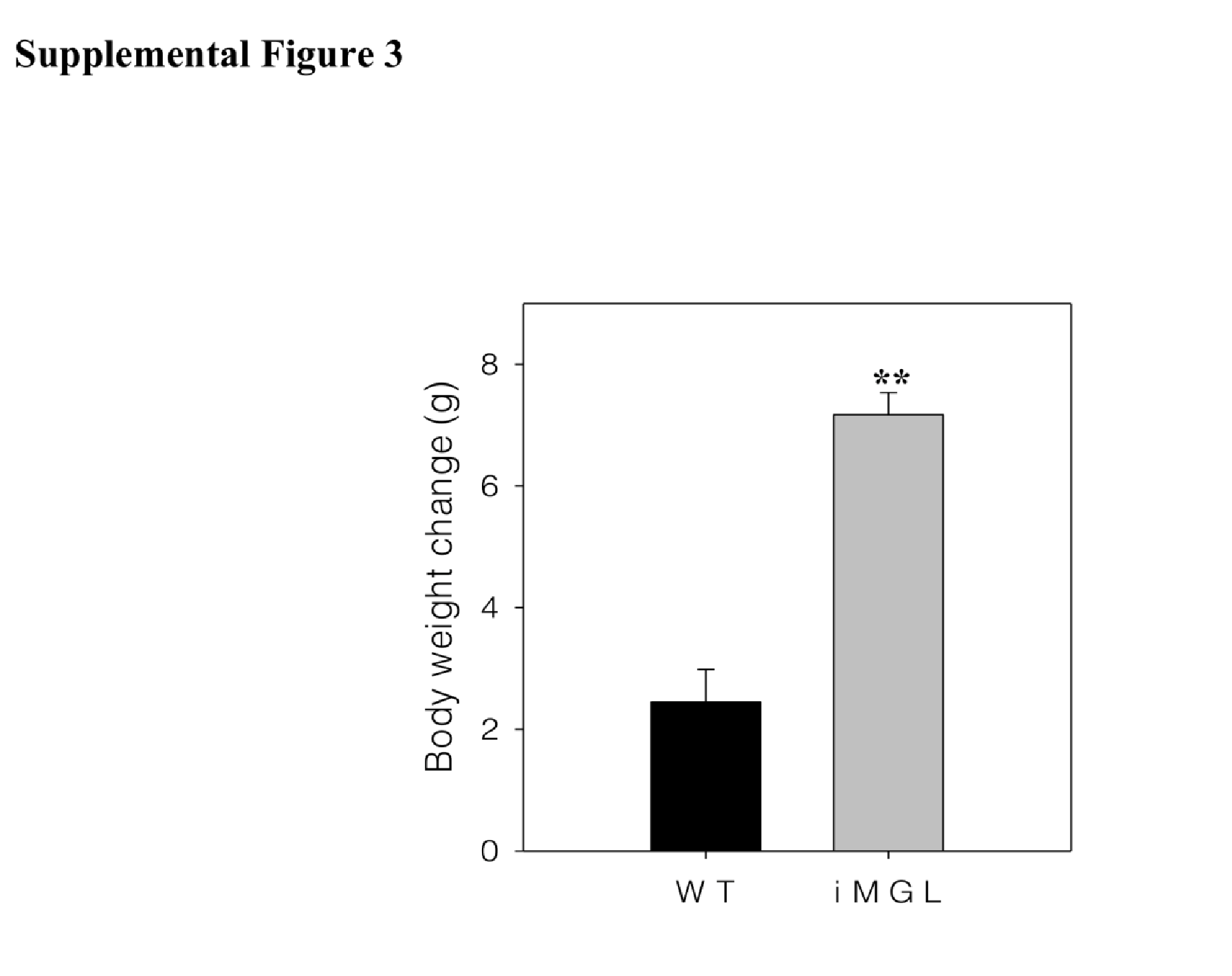

Supplement: Figure S3 — Body weight changes in 361-1 line iMGL mice after 3 weeks of a high fat (40% kcal) diet. F4 generation mice backcrossed with SJL (WT; n = 3, iMGL; n = 4). Data represent mean ± S.E. ** p<0.01 versus wild type littermates. (TIF) [file pone.0043962.s003.tif]

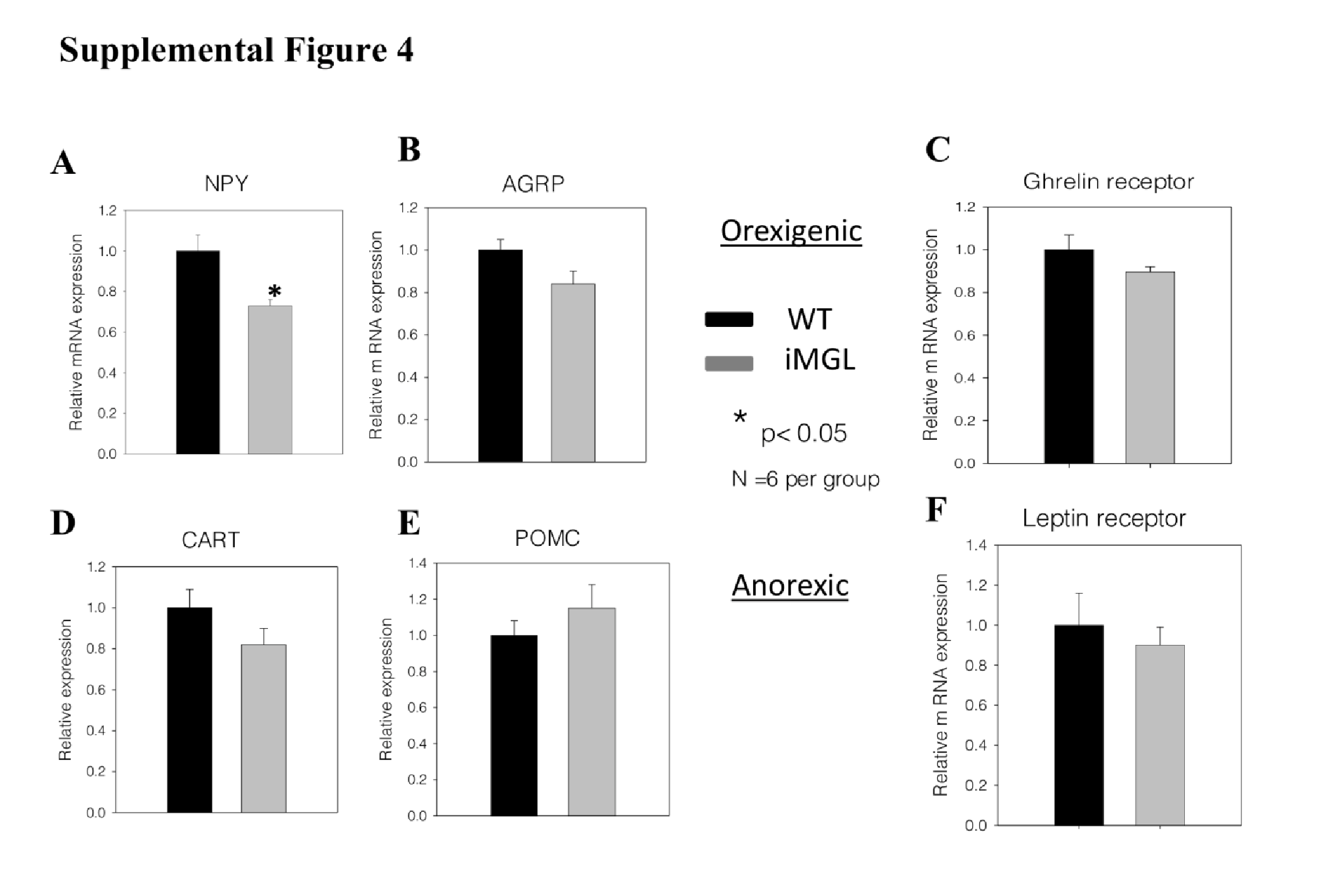

Supplement: Figure S4 — Brain neuropeptide mRNA expression in iMGL mice by QPCR analysis. (A) Neuropeptide Y (NPY), (B) Agouti-related protein (AGRP), (C) Ghrelin receptor (D) Pro-opiomelanocortin (POMC), (E) Cocaine and amphetamine regulated transcript (CART), and (F) Leptin receptor. Values are presented relative to the expression of the wild type littermates set to 1. Data represent mean ± S.E. * p<0.05 versus wild type littermates (n = 6 per group). (TIF) [file pone.0043962.s004.tif]
