# Supplementary material for: Over-Expression of Monoacylglycerol Lipase (MGL) in Small Intestine Alters Endocannabinoid Levels and Whole Body Energy Balance, Resulting in Obesity
Source: PLoS One. 2012 Aug 28;7(8):e43962. doi: 10.1371/journal.pone.0043962 (PMC3429419; doi:10.1371/journal.pone.0043962)
Supplement: Table S1 — Plasma parameters in iMGL mice. Plasma TG, cholesterol, fatty acid, 2-AG, glucose and insulin levels (n = 8∼16), gut peptides (active ghrelin and total PYY) (n = 4), and AEA, OEA, and NAPEs (2 pooled samples from total of n = 8 mice per group). Data represent mean ± S.E. * p<0.05 versus wild type littermates. (DOC) [file pone.0043962.s005.doc]

|  | | **Wild Type** | **iMGL** |
| --- | --- | --- | --- |
| **TG**  (mg/dL) | Fed | 79.7 ± 5.9 | 104.6 ± 10.4***** |
|  | Fasting | 45.7 ± 5.3 | 34.2 ± 1.6 |
| **Cholesterol**  (mg/dL) | Fed | 178.2 ± 11.4 | 178.3 ± 8.3 |
|  | Fasting | 114.7 ± 11.3 | 113.3 ± 10.9 |
| **Fatty acid**  (mM) | Fed | 2.74 ± 0.18 | 2.51 ± 0.07 |
|  | Fasting | 0.83 ± 0.20 | 0.79 ± 0.23 |
| **Insulin**  (ng/mL) | Fed | 0.30 ± 0.07 | 0.46 ± 0.13 |
|  | Fasting | 0.24 ± 0.05 | 0.31 ± 0.04 |
| **Glucose**  (mg/dL) | Fasting | 159.4 ± 7.8 | 146.8 ± 9.9 |
| **2-AG**  (ng/mL) | Fed | 39.5 ± 7.0 | 44.7 ± 8.0 |
|  | Fasting | 50.6 ± 10.4 | 30.9 ± 6.8 |
| **AEA**  (ng/mL) | Fasting | 0.77, 0.95 | 0.20, 0.63 |
| **(C16:0) NAPE**  (ng/mL) | Fasting | 59.6, 57.3 | 29.7, 24.8 |
| **(C18:1)NAPE**  (ng/mL) | Fasting | 36.2, 36.4 | 23.6, 20.7 |
| **(C22:4)NAPE**  (ng/mL) | Fasting | 32.9, 37.4 | 21.9, 20.1 |
| **OEA****  **(ng/mL)** | Fasting | 1.28, 2.30 | 1.08, 1.95 |
| **PYY**  (pg/mL) | Fasting | 42.3 ± 6.2 | 41.0 ± 14.4 |
| **Ghrelin**  (pg/mL) | Fasting | 11.7 ± 3.0 | 5.7 ± 0.9 |
