# Supplementary material for: Over-Expression of Monoacylglycerol Lipase (MGL) in Small Intestine Alters Endocannabinoid Levels and Whole Body Energy Balance, Resulting in Obesity
Source: PLoS One. 2012 Aug 28;7(8):e43962. doi: 10.1371/journal.pone.0043962 (PMC3429419; doi:10.1371/journal.pone.0043962)
Supplement: Table S2 — QPCR primer sequences. (DOC) [file pone.0043962.s006.doc]

| **β-Actin** | Forward 5’-GGC TGT ATT CCC CTC CAT CG-3’  Reverse 5’-CCA GTT GGT AAC AAT GCC ATG T-3’ |
| --- | --- |
| **MGAT1** | Forward 5’-TGTCTTGTCAAAACGCAGGAT-3’  Reverse 5’-ACAACGGGAAACAGAACCAGA-3’ |
| **MGAT2** | Forward 5’-TGG GAG CGC AGG TTA CAG A-3’  Reverse 5’-CAG GTG GCA TAC AGG ACA GA-3’ |
| **GPAT3**  **(ER GPAT )** | Forward 5’-TAT CCA AAG AGA TGA GTC ACC CA-3’  Reverse 5’-CAC AAT GGC TTC CAA CCC CTT-3’ |
| **GPAT1** | Forward 5’-CTGCTTGCCTACCTGAAGACC-3’  Reverse 5’-GATACGGCGGTATAGGTGCTT-3’ |
| **CPT1** | Forward 5’-AGCACACCAGGCAGTAGCTT-3’  Reverse 5’-AGGATGCCATTCTTGATTCG-3’ |
| **ACOX1** | Forward 5’-ATATTTACGTCACGTTTACCCCGG-3’  Reverse 5’-GGCAGGTCATTCAAGTACGACAC-3’ |
| **FASN** | Forward 5’-AGGTGGTGATAGCCGGTATGT-3’  Reverse 5’-TGGGTAATCCATAGAGCCCAG-3’ |
| **ACC1**  **(ACACA)** | Forward 5’-ATGGGCGGAATGGTCTCTTTC-3’  Reverse 5’-TGGGGACCTTGTCTTCATCAT-3’ |
| **ATGL** | Forward 5’-GGTCCTCCGAGAGATGTGC-3’  Reverse 5’-TGGTTCAGTAGGCCATTCCTC-3’ |
| **HSL** | Forward 5’-TTCTCCAAAGCACCTAGCCAA-3’  Reverse 5’-TGTGGAAAACTAAGGGCTTGTTG-3’ |
| **DGAT1** | Forward 5’-TGT TCA GCT CAG ACA GTG GTT-3’  Reverse 5’-CCA CCA GGA TGC CAT ACT TGA T-3’ |
| **DGAT2** | Forward 5’-TTC CTG GCA TAA GGC CCT ATT-3’  Reverse 5’-AGT CTA TGG TGT CTC GGT TGA C-3’ |
| **PPARγ** | Forward 5’-TGTGGGGATAAAGCATCAGGC-3’  Reverse 5’-CCGGCAGTTAAGATCACACCTAT-3’ |
| **PPARα** | Forward 5’-TCG GCG AAC TAT TCG GCT G-3’  Reverse 5’-GCA CTT GTG AAA ACG GCA GT-3’ |
| **FAAH** | Forward 5’-GAGGCTCCCCTCTGGGTTTA-3’  Reverse 5’-GCCAGGCTATCCACATCCC-3’ |
| **MGL**  **(Coding region)** | Forward 5’-CAGAGAGGCCAACCTACTTTTC-3’  Reverse 5’-ATGCGCCCCAAGGTCATATTT-3’ |
| **MGL**  **(3’UTR)** | Forward 5’-CCTTAAGGAGCAGGCACTTTATG-3’  Reverse 5’-TCAAAGGATATTTCCACACAGTCTCT-3’ |
| **MGL+SV40** | Forward 5’-GGAGTCAGGACAAAACACTCAAGATGTA-3’  Reverse 5’-ACTAGATGGCATTTCTTCTGAGCAAAAC-3’ |
| **CB1** | Forward 5’-GGG CAC CTT CAC GGT TCT G-3’  Reverse 5’-GTG GAA GTC AAC AAA GCT GTA GA-3’ |
| **Leptin** | Forward 5’-TGACACCAAAACCCTCATCA-3’  Reverse 5’-AGCCCAGGAATGAAGTCCA-3’ |
| **Leptin**  **Receptor** | Forward 5’-GTG CTA ACT TCT CTG GGT CTA A-3’  Reverse 5’-CAG GAC TAT GGA TAA ACC CTT GC-3’ |
| **Ghrelin**  **Receptor** | Forward 5’-TGG AGA TCG CGC AGA TCA G-3’  Reverse 5’-CCG GGA ACT CTC ATC CTT CAG-3’ |
| **POMC** | Forward 5’-CTG GAG ACG CCC GTG TTT C-3’  Reverse 5’-TGG ACT CGG CTC TGG ACT G-3’ |
| **AGRP** | Forward 5’-ATG CTG ACT GCA ATG TTG CTG-3’  Reverse 5’-CAG ACT TAG ACC TGG GAA CTC T-3’ |
| **NPY** | Forward 5’-ATG CTA GGT AAC AAG CGA ATG G-3’  Reverse 5’-TGT CGC AGA GCG GAG TAG TAT-3’ |
| **CART** | Forward 5’-GCT CAA GAG TAA ACG CAT TCC G-3’  Reverse 5’-ACA AGC ACT TCA AGA GGA AAG AA-3’ |
